# Further content validation of the 18-item NCCN/FACT Ovarian Symptom Index and its Disease Related Symptom-Physical (DRS-P) subscale for use in advanced ovarian cancer clinical trials

**DOI:** 10.1186/s12955-019-1253-3

**Published:** 2019-12-19

**Authors:** Sara Shaunfield, Sally Jensen, Allison P. Fisher, Kimberly Webster, Shohreh Shahabi, Arijit Ganguli, David Cella

**Affiliations:** 1 Department of Medical Social Sciences, Northwestern University Feinberg School of Medicine, 625 N. Michigan Ave. Suite 2700, Chicago, IL 60611 USA; 2 Department of Obstetrics and Gynecology, Division of Gynecologic Oncology, Robert H. Lurie Comprehensive Cancer Center, Northwestern University Feinberg School of Medicine, Chicago, IL USA; 3 AbbVie Inc., North Chicago, IL USA

**Keywords:** Advanced ovarian cancer, Patient reported outcomes, Symptom index, Clinical trials, Content validation, Qualitative methods

## Abstract

**Background:**

This study evaluated pre-defined aspects of content validity of the 18-item NCCN FACT-Ovarian Symptom Index (NFOSI-18) and its Disease-Related Symptoms-Physical (DRS-P) subscale, as clinical trial outcome tools for patients with advanced ovarian cancer.

**Methods:**

Twenty-one women (mean age 59.5 years) diagnosed with advanced ovarian cancer completed the NFOSI-18 and participated in a cognitive interview to explore: (1) whether ‘pain’ and ‘cramps’ are considered redundant; (2) whether ‘fatigue’ and ‘lack of energy’ are overlapping concepts; (3) whether patients consider severity when responding to the item “I am bothered by constipation;” and (4) factors considered when responding to the item “I am sleeping well.” Interviews were audio-recorded, transcribed, and analyzed qualitatively.

**Results:**

Pain was associated with discomfort, hurt, and life interference; ‘cramps’ was associated with pain, muscle tightening, and menstrual or digestive issues. Most (81%) considered the items “I have pain” and “I have cramps in my stomach area” to be more different than similar. Participants associated ‘fatigue’ with intense tiredness and ‘lack of energy’ with motivation and capability to complete daily activities. Item comparisons revealed a majority (65%) considered the items to be more different than similar. When responding to “I am bothered by constipation,” patients indicated constipation severity was related to bother. Finally, patients considered disease, treatment, and other factors when responding to “I am sleeping well.”

**Conclusions:**

Findings support content validity of the NFOSI-18 and its DRS-P as originally constructed. We propose an alternative scoring option that excludes the item “I am sleeping well” from the DRS-P when used as a symptom-focused index for clinical research in a regulatory context.

## Introduction

Ovarian cancer represents the second most common and deadliest gynecologic cancer [1]. Due to lack of detectable early symptoms and effective screening measures, over 60% of ovarian cancer cases are diagnosed at an advanced stage. Women with advanced disease experience a number of disease- and treatment-related symptoms and concerns impacting quality of life (QOL), including fatigue, pain, swelling, nausea, vomiting, as well as the emotional and psychological burden of living with the disease [2]. The limited options for cure among women with advanced disease, coupled with the treatment and disease burden they face, highlights the significance of QOL as an important clinical and research outcome.

The NCCN-FACT Ovarian Symptom Index-18 (NFOSI-18) was developed to provide a clinically meaningful patient-reported symptom index reflecting the symptoms and concerns identified as most important by women with advanced ovarian cancer [3, 4]. Four subscales comprise the 18-item index: disease-related symptoms-physical (DRS-P; 9 items), disease-related symptoms-emotional (1 item), treatment side effects (5 items), and general function/well-being (3 items). The recall period is the past 7 days [5]. The NFOSI-18 has demonstrated good preliminary reliability and validity [2].

The U.S. Food and Drug Administration (FDA) emphasizes improvement in tumor related symptoms, or delay in symptom progression, as potential evidence of clinical benefit for patients in oncology drug trials. Patient-reported outcome (PRO) measures such as the NFOSI-18, and its 9-item DRS-P subscale, can serve as useful tools to assess symptoms in clinical trials and practice. When evaluating whether PRO measures meet regulatory standards for use in clinical trials, the FDA places high priority on evidence supporting content validity [3]. As part of ongoing efforts to examine the content validity of the NFOSI-18, a recent study found that 89% of participants reported the items were clear and understandable, and 89% reported that they were either “very confident” or “confident” in their ability to respond to 17 of 18 items [3]. In response to four questions raised during regulatory review, and as the next step in the validation process, we sought to further examine the content validity of the NFOSI-18, and its 9-item DRS-P subscale, for use in clinical research in a regulatory context. The NFOSI-18 can be found at FACIT.org [5]. We explored the following four specific questions: (1) Do patients consider ‘pain’ and ‘cramps’ to be redundant concepts?; (2) Are ‘fatigue’ and ‘lack of energy’ overlapping concepts?; (3) Does the item “I am bothered by constipation” assess severity?; (4) What factors do patients consider when responding to: “I am sleeping well?”

## Methods

### Design

We conducted a qualitative study with women diagnosed with advanced ovarian cancer recruited from the Division of Gynecologic Oncology at Northwestern Medicine who completed the NFOSI-18 and then participated in a cognitive interview to assess the specific study questions. Interviews were conducted between February and April of 2017. The study was approved by the Northwestern University Institutional Review Board (STU00203656) and informed consent was obtained for all participants.

### Participants

Patients were eligible to participate if they met the following criteria: (a) ≥18 years of age; (b) diagnosis of stage III or IV high-grade serous adenocarcinoma of epithelial ovarian, fallopian tube, or primary peritoneal cancer; (c) ECOG performance status = 0–2; (d) about to begin treatment or received treatment for ovarian cancer within the past 12 months; and (e) fluent in English. A trained interviewer approached eligible patients, explained the study, and obtained consent. Patients were compensated $50 for their time and effort.

### Procedures

**Fig. 1:**
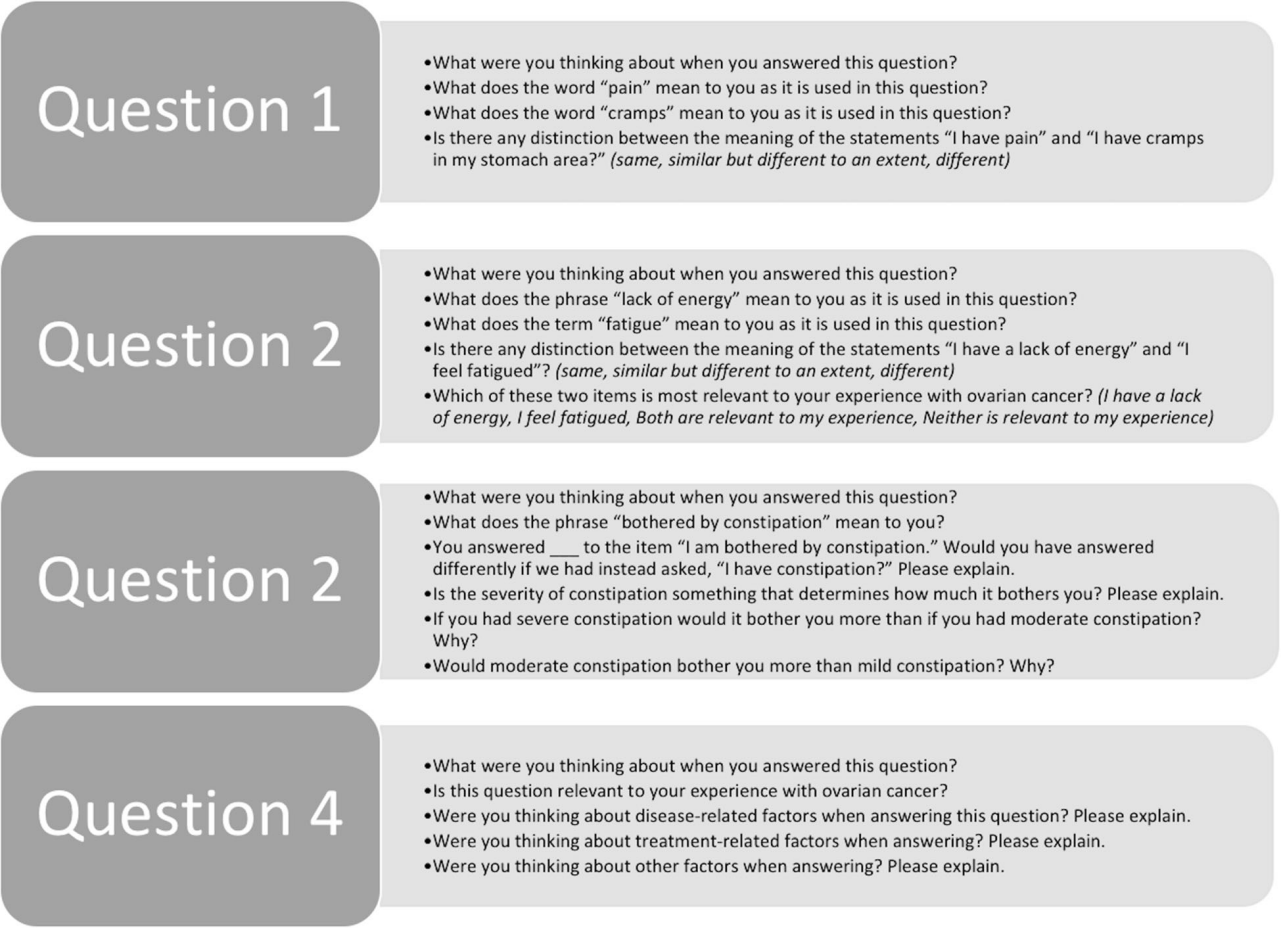
Cognitive interview questions targeted to the four study questions

Cognitive interviews were conducted in-person and were audio recorded. Trained researchers used a semi-structured interview guide modeled after guides used in prior qualitative work to assess content validity [6–9]. The guide was tailored to elicit targeted participant responses in accordance with the four study questions. First, participants provided basic sociodemographic information including disease and treatment history. Patients then completed the NFOSI-18 [3]. Next, the interviewer reviewed each NFOSI-18 item and asked participants a series of questions to assess content validity of the DRS-P and targeted probes to ascertain detailed responses regarding the specific study questions. Figure 1 illustrates the targeted probes tailored to elicit responses according to each question.

### Data analysis

Interview transcripts were analyzed via constant comparative approach [10, 11]. First, an initial codebook was developed using detailed interviewer field notes. Next, three transcripts were independently reviewed and coded in Dedoose [12], a cross-platform application for qualitative analysis, by trained qualitative researchers. Each analyst made notes about missing or problematic codes. The group met and reviewed the coded transcripts, discussed discrepancies, and edited the codebook. Once the final codebooks were constructed, remaining transcripts were divided among two team members for independent coding. After coding was completed, text for each code was extracted and reviewed in a ‘coding review process,’ during which codes were collapsed into overarching themes and summarized. This process was conducted separately for each of the four study questions. Responses to categorical interview questions were summarized and used to confirm emergent themes. Summaries of the qualitative themes for each study question, and findings from the categorical interview questions constituted the data used to evaluate the content validity of the NFOSI-18 DRS-P as a targeted symptom index for clinical research in a regulatory context.

## Results

### Participants

Twenty-one participants completed cognitive interviews. Most (76%) were Caucasian, with a mean age of 59.5 years. A majority (*n* = 19, 90%) had a self-reported ECOG status ≥1. On average, women were diagnosed 1.3 years prior to the interview. At the time of the interview, most (72%) were currently receiving treatment, and over half (*n* = 13, 62%) underwent cytoreductive surgery. Of the 13 who had cytoreductive surgery, most (*n* = 10, 77%) had neoadjuvant chemotherapy followed by interval debulking while a few underwent cytoreductive surgery prior to beginning chemotherapy (*n* = 3, 23%). Tables 1 and 2 detail patient demographics and clinical characteristics.

**Table 1:** Demographic characteristics of cognitive interview sample (*N* = 21)

Characteristic	n (%)
**NFOSI-18 Score**^**1**^	**Mean (range, median)**
DRS-P subscale	25 (9–31, 27)^2^
Total score	52.5 (31–60, 56)^3^
**Age**	59.5 (39–73, 57)
**Education**	
Eighth grade or less	1 (5%)
High school	7 (33%)
Some college	10 (48%)
College	2 (9%)
Advanced degree	1 (5%)
**Ethnicity**	
Hispanic or Latino origin	2 (9.5%)
Non-Hispanic or Latino origin	19 (90.5%)
**Race**	
White	16 (76%)
African American or Black	3 (14%)
Asian	1 (5%)
Other (biracial)	1 (5%)
**Marital Status**	
Never married	2 (9.5%)
Married	11 (52%)
In a committed relationship	1 (5%)
Divorced	5 (24%)
Widowed	2 (9.5%)
**Employment Status**	
Employed full-time	9 (43%)
Employed part-time	2 (9.5%)
Homemaker	1 (5%)
Unemployed	1 (5%)
Retired	2 (9.5%)
On disability	4 (19%)
On leave of absence	2 (9.5%)

^1^Lower scores indicate greater symptom burden (i.e., poorer quality of life)

^2^DRS-P subscale score (range 0–36)

^3^NFOSI-18 total score range (range 0–72)

**Table 2:** Clinical characteristics of cognitive interview sample (N = 21)

Characteristic	n (%)
**Years since diagnosis**	***Mean (range, median)***
	1.33 (0.03–9, 0.5)
**ECOG Status**^**1**^ **(Self-reported)**	
0	2 (10%)
1	11 (52%)
2	8 (38%)
**Diagnosis**	
Ovarian	13 (62%)
Primary Peritoneal	4 (19%)
Ovarian and Fallopian Tube	3 (14%)
Fallopian Tube	1 (5%)
**Cytoreductive surgery**	
Yes	13 (62%)
No	8 (38%)
**Treatment (Tx) status at the time of interview**	
1st line Tx	9 (43%)
Receiving Tx for a recurrence	6 (29%)
Tx naive	3 (14%)
Received Tx within past 12 months	3 (14%)
**Neoadjuvant chemotherapy**	
No	11 (52%)
Yes	10 (48%)

^1^0 = “Fully active, able to carry on all pre-disease performance without restriction”; 1 = “Restricted in physically strenuous activity but ambulatory and able to carry out work of a light or sedentary nature”; 2 = “Ambulatory and capable of all selfcare but unable to carry out any work activities” [13]

### Question 1: Do patients consider ‘pain’ and ‘cramps’ to be redundant concepts?

#### Patient interpretation of ‘pain’

Our analysis of patient interpretations of pain when responding to the item “I have pain” revealed three prominent themes including discomfort, hurt, and interference. When describing the meaning of pain, 7 (33%) defined it as a form of *discomfort* and several specified that pain is discomfort that is extreme, noticeable, or physical. For example, when asked how she defines the meaning of pain, Pt 018 responded, “extreme discomfort.” Similarly, Pt 001 described pain as, “discomfort to a noticeable level which varies with different people.” On the other hand, 5 (24%) used the term *hurt* when defining ‘pain’, like Pt 010 who explained, “pain means something that hurts and does not feel good and can cause discomfort.” While hurt and discomfort were often used interchangeably, one patient emphasized that ‘pain’ means “hurting above discomfort” (Pt 005). Finally, 5 (24%) described pain in terms of *interference* with daily life or life quality. For example, Pt 011 defined pain as “a kind of constant feeling that is disrupting my quality of life.” Furthermore, patients reported considering various forms of pain when responding to the item “I have pain,” including joint or bone pain, abdominal pain or discomfort, side or lower back pain, and occasionally women recalled pain associated with treatment (i.e., neuropathy, skin problems, mouth sores, post-operative pain).

#### Patient interpretation of ‘cramps’

When defining the meaning of cramps as used in the item “I have cramps in my stomach area” women referenced concepts such as pain/discomfort, muscle tightening, menstrual cramps, or digestive issues. Over half (*n* = 14, 67%) defined ‘cramps’ as *pain/discomfort*. For example, Pt 009 conceptualized it as “…an aching in the stomach. I don’t know severe pain.” Meanwhile, Pt 001 described ‘cramps’ as “kind of a pain that isn’t sharp, it is dull in a debilitating radiating feeling.” While a majority of patients referenced pain/discomfort when defining the meaning of cramps, many spontaneously differentiated cramps from pain by describing ‘cramps’ as a subset of pain. For example, Pt 015 explained, “[it’s] like a very specific passing pain. That’s like deep in your abdomen…like you can tell [it is] a muscle pain.” Terms used to describe the pain associated with ‘cramps’ were shooting, sharp, debilitating, radiating, aching, severe, slight, passing, dull, and tenderness. In addition to pain/discomfort, nearly half (*n* = 10, 48%) defined cramps as *muscle tightening*, a gripping feeling or tightening of the muscles. For example, Pt 010 shared, “cramps are a pain that feels like my stomach in a certain area or whatever area is cramping is balling up…preventing me from breathing comfortably.”

In addition to patient interpretations of cramps as related to pain, discomfort, and muscle tightening, a subset (*n* = 7; 33%) likened the sensation to that of *menstrual cramps*. For example, Pt 001 shared, “at times I have felt the same kind of cramping as when I had menstrual cramps…I thought I shouldn’t be having these anymore…does that mean something because I am feeling cramping when I don’t have a uterus?” Similarly, Pt 013 elaborated, “When you have cramps, they can be very painful, you know it’s like having periods. The cramps of your period. They are painful, but we call them cramps. It’s like a sub-category.” Additionally, when asked to define the meaning of cramps, a few noted that as women, they instinctively think of menstrual cramps, like Pt 011, “…as woman I automatically think of menstrual cramps, and what I feel now is very different…so that’s why I was like, ‘Do you mean pain or like that crampy, like that sort of clinching consistent feeling?’”. On the other hand, 6 (29%) considered *digestive issues* such as diarrhea, constipation, or gas when responding to the item or defining ‘cramps.’ While several reported experiencing cramps as a symptom prior to diagnosis and treatment, for most, the symptom subsided following treatment.

While there was some overlap in patient interpretation of ‘pain’ and ‘cramps’, distinctions also emerged. As illustrated in Table 3, a comparison of emergent themes regarding patient interpretation of ‘pain’ and ‘cramps’ reveals both similarities and differences. Specifically, women conceptualized ‘pain’ as interfering with one’s life whereas ‘cramps’ were considered a subset of pain involving muscle tightening. While the concepts are related, women clearly distinguished the two. Further, investigation into potential item redundancy revealed most (*n* = 17, 81%) women considered the items to be “different” or “similar but different”, and many elaborated that the item “I have cramps in my stomach area” refers to a targeted area, whereas “I have pain” is broad and could encompass various forms of pain. For example, Pt 003 explained “because you can have pain in your joints or in your muscles where the other one is a specific area…I think they’re *completely* different.”

**Table 3:** Comparison of emergent themes regarding patient interpretations of ‘pain’ and ‘cramps’

	Themes						
Concepts	Pain	Discomfort	Hurt	Interference	Digestion	Menstrual Cramps	Muscle Tightening
Pain	X	X	**X**	**X**			
Cramps	X	X			**X**	**X**	**X**

### Question 2: Are ‘fatigue’ and ‘lack of energy’ overlapping concepts?

#### Patient interpretation of ‘fatigue’

Our exploration of patient interpretation of ‘fatigue,’ as used in the item “I feel fatigued”, revealed five prominent conceptualizations of ‘fatigue’ including tiredness, intensity, energy, rest, and comparison.

Most women (n = 19, 90%) defined ‘fatigue’ in terms of one’s level of *tiredness,* which reflects feeling or being tired, as described by Pt 014, a “general overall tiredness.” Moreover, 9 (43%) defined the quality of ‘fatigue’ in terms of *intensity*, describing fatigue as another level of tiredness or an extreme version of exhaustion, using qualifiers such as “overwhelming tiredness” (Pt 019), “total exhaustion” (Pt 018), being “very, very tired” (Pt 021), and “[fatigue is] that one more step, [an] extreme” (Pt 005). Others described the intensity of ‘fatigue’ as “fatigue takes over” (Pt 015) or “I don’t even feel like I can move” (Pt 013). Furthermore, 8 (38%) conceptualized ‘fatigue’ in terms of one’s *energy* level, specifically having a lack of or no energy at all. As Pt 002 explained, “lack of energy, you know just feeling tired and lack of energy and wanting to nap.” Moreover, a subset (*n* = 6, 29%) qualified ‘fatigue’ as something that requires *rest*, the need to sit or lie down, or a need for sleep. As Pt 001 explained, “being too tired to accomplish a task without resting.” Some (n = 6, 29%) framed their current level of fatigue in *comparison* to a previous state using qualifiers such as “more than I would normally [have]” (Pt 006), “compared to how I was on chemo” (Pt 016), and “before I got sick” (Pt 008).

#### Patient interpretation of ‘lack of energy’

Our investigation into patient interpretations of ‘lack of energy’ when responding to the item “I have a lack of energy” revealed five prominent ways that patients conceptualize the concept, including energy, tiredness, capability, motivation, and comparison.

Patients most commonly (*n* = 15, 71%) defined ‘lack of energy’ in terms of one’s *energy* level, specifically having little energy or no energy at all. For example, Pt 010 shared, “I don’t feel energetic enough or upbeat enough to perform my daily usual daily routines.” On the other hand, 9 (43%) interpreted the phrase in terms of the presence of *tiredness,* specifically feeling or being “tired” or “wiped out.” Moreover, nearly half (*n* = 10; 48%) defined ‘lack of energy’ in terms of their *capability*, or the impact that low energy has on one’s ability to perform routine tasks or exercise. For example, Pt 018 defined it as “not being able to take a walk or get up and do routine household stuff.” Alternatively, women used terms indicative of *motivation,* as described by Pt 017 that “having a lack of energy means not feeling like doing things.” Specifically, 9 (43%) defined the phrase as having “no motivation”, “inspiration”, or “desire” to begin or complete a task or action, such as getting up from the couch or going to the grocery store. Finally, 8 (38%) defined the phrase by making a *comparison* between their current energy level and how they felt at another point in time, such as before having cancer.

As shown in Table 4, a comparison of emergent themes regarding how patients conceptualize ‘fatigue’ and ‘lack of energy’ reveals both similarities and differences. For example, *tiredness* and *energy* are interpretive themes for both concepts. Moreover, comparison of the themes across items reveals distinctions. Specifically, patients conceptualize ‘lack of energy’ as involving a mental component suggesting the item taps into motivation, as well as one’s capability to achieve activities of daily living. Fatigue, on the other hand, was conceptualized as tiredness that was greater in intensity or more extreme than ‘lack of energy,’ and is indicative of the need for rest or sleep. This subtle distinction was described by Pt 019:

[Fatigue is] a feeling of overwhelming tiredness. Even little tasks tire me. A lack of energy…is related to motivation, so if I wake up in the morning and don’t want to do anything. That…is lack of energy. If I do a few things and I’m tired…that [is] fatigue.

Moreover, our investigation into potential item redundancy revealed that a majority (*n* = 16, 76%) considered the items to be “different” or “similar but different.”

**Table 4:** Comparison of emergent themes regarding patient conceptualizations of ‘fatigue’ and ‘lack of energy’^1^

	Themes						
Concepts	Tiredness	Energy	Comparison	Intensity	Rest	Capability	Motivation
Fatigue	X	X	X	**X**	**X**		
Lack of energy	X	X	X			**X**	**X**

#### Question 3: Does the item “I am bothered by constipation” assess severity?

A majority (n = 16, 76%) indicated constipation severity influenced how much constipation bothered them (Table 5). Of the 11 who endorsed the item “I am bothered by constipation,” nearly all reported they would be more bothered if the constipation worsened, and 100% said they would be less bothered if the constipation improved. Further, some explained they would be more bothered if interventions such as stool softeners did not work, if constipation was more frequent and/or more painful, if constipation episodes lasted longer, and if constipation caused more spasms. For example, Pt 007, explained, “Just greater levels of discomfort…stronger cramps, longer periods without bowel movements.” On the other hand, patients suggested they would be less bothered if constipation caused less pain or was less frequent. Taken together, these findings indicate patients do consider the severity of constipation when responding to the item “I am bothered by constipation.”

**Table 5:** Patient consideration of severity when responding to the item “I am bothered by constipation” (*N* = 21)

Item	Is the severity of constipation something that determines how much it bothers you?	Item endorsement *(Patients who selected 0* vs *1–4)*^1^	Imagine your constipation getting worse, would you be more bothered by it? (*n* = 11)^2^	Imagine your constipation getting better, would you be less bothered by it? (n = 11)^2^
I am bothered by constipation	Yes = 16 (76%)	0 = 10 (48%)	Yes = 9 (82%)	Yes = 11 (100%)
	No = 2 (10%)	1–4 = 11 (52%)	No = 1 (9%)	No = 0 (0%)
	Missing = 3 (14%)^3^		Missing = 1 (9%)^3^	

^1^0 = Not at all; 1 = A little bit; 2 = Somewhat; 3 = Quite a bit; 4 = Very much

^2^Question asked only to those who selected “A little Bit,” “Somewhat,” “Quite a Bit,” or “Very Much”

^3^Missing indicates unclear response or no response

### Question 4: What factors do patients consider when responding to: “I am sleeping well?”

Although a majority (*n* = 15, 71%) reported the item “I am sleeping well” as relevant to their experience with ovarian cancer, several notable themes related to sleep quality emerged. Patients described consideration of their ovarian cancer experience when responding to this item in three ways, including disease-related factors affecting sleep, treatment-related factors affecting sleep, and emotional concerns affecting sleep (Table 6). Other non-cancer-related factors affecting sleep also emerged during analysis.

**Table 6:** Factors patients consider when responding to the item “I am sleeping well” (*N* = 21)

Item	Is this question relevant to your experiences with ovarian cancer?	Were you thinking of disease-related factors when answering this question?	Were you thinking about treatment-related factors when answering?	Were you thinking about other factors when answering?
I am sleeping well	Yes = 15 (71%)	Yes = 11 (52%)	Yes = 12 (57%)	Yes = 7 (33%)
	No = 6 (29%)	No = 10 (48%)	No = 5 (24%)	No = 14 (67%)
			N/A = 4 (19%)^1^	

^1^N/A = Treatment naïve patients were not asked this question

#### Ovarian cancer-related factors affecting sleep

A subset (*n* = 3, 14%) considered *disease*-related factors when responding to the item. Although they did not identify specific disease processes affecting sleep, women attributed sleep disturbances to the experience of having ovarian cancer, in general. For example, Pt 003 stated, “…it’s everything combined. In the back of my mind you still hear that Stage IV ovarian cancer diagnosis.”

Nearly half (*n* = 10, 48%) stated that their sleep was affected by factors related to *treatment*. A subset of patients cited treatment, in general, as negatively impacting sleep. Patients also specifically described chemotherapy as a factor affecting sleep quality. For example, Pt 003 shared, “usually two days after chemo I don’t sleep and that’s what bothers me.” In addition to the effect of chemotherapy on sleep, patients also described thinking about other cancer-related therapies when responding, as Pt 007 explained, “I take steroids on Sunday…and I think it’s very directly related to the treatment and not stress or anything like that…because it’s very clearly Mondays, Sundays…[are] nights I might have disrupted sleep.”

On the other hand, a subset (*n* = 7, 33%) considered the *emotional* impact when responding to the item, “I am sleeping well,” citing anxiety, worry, racing mind, and depression when describing emotional interference with sleep quality. Although some described anxiety affecting sleep generally, others specifically stated that ovarian cancer-related anxiety and having an uncertain future affected sleep. For example, Pt 008 explained, “I think that in my case it’s a concern that is coming next, it’s interesting how this comes because I think one of my difficulties in sleeping is I think too much about what’s gonna’ happen to me.”

#### Other factors affecting sleep

In addition to the physical and emotional consequences of ovarian cancer and its treatment, a subset of patients (n = 7, 33%) reported considering factors unrelated to having ovarian cancer when responding to this item. Several described a longstanding history of sleep difficulty predating their cancer experience, as demonstrated by Pt 002, “I had problems with sleeping before [cancer]…I don’t know some age thing or what, but I can’t say it’s really connected…[my] sleeping issue didn’t happen as a result of my cancer or my chemo.” Patients also described sleep interference related to non-cancer medical conditions, such as hot flashes and need to use the restroom at night.

Finally, when asked whether the item was related to disease-related symptoms, treatment-related side effects, or other factors,^1^ a relatively equal number responded that the item was related to disease-related factors (*n* = 11; 52%) and treatment-related factors (*n* = 12; 57%).

## Discussion

This study provides further evidence for the validity of the NFOSI-18 and its 9-item DRS-P for use in clinical research within a regulatory context. Specifically, we sought to further evaluate the content validity of these instruments to explore: (1) whether patients consider ‘pain’ and ‘cramps’ as to be redundant concepts; (2) whether ‘fatigue’ and ‘lack of energy’ are overlapping concepts; (3) whether the item “I am bothered by constipation” assesses severity; and (4) factors patients consider when responding to the item “I am sleeping well.” The results of this study provide further support for the content validity of the NFOSI-18 DRS-P as appropriate for use among women diagnosed with advanced ovarian cancer.

Our findings provide qualitative insight into patient interpretations of key item content that assess related yet distinct concepts. First, our investigation into whether women with advanced ovarian cancer consider pain and cramps to be redundant, revealed that women consider cramps to be a subset of pain. Moreover, while there was some overlap in the way patients described pain and cramps as a form of discomfort, key differences emerged. For example, patients distinguished cramps from pain through depictions of muscle tightening. Moreover, similar to prior content validity findings [3], patients referenced digestive issues or menstrual cramps in their descriptions of cramps. While these findings clearly illuminate distinctions, when directly asked whether the items were measuring the same concepts, a vast majority considered the concepts to be similar but different or altogether different. Thus, results of question 1 suggest that pain and cramps are distinct and relevant concepts, and both items should be retained on the NFOSI-18 DRS-P subscale.

Experts and patients alike report fatigue as the most important symptom for advanced cancer patients who have undergone chemotherapy and therefore it is vital to accurately capture the symptom on PROs and symptom indices [14]. In light of its significance, we sought to explore whether the items ‘I have a lack of energy’ and ‘I feel fatigued’ are measuring the same concept and could be considered redundant. In the present study, some overlap emerged in patient descriptions of the two concepts; however, clear distinctions emerged between the two. Specifically, patients defined fatigue as an intense need for rest, whereas lack of energy was conceptualized in terms of one’s motivation or capability to initiate or accomplish daily activities. These results are in line with findings from a recent content validation study in that the item “I have a lack of energy” is related to activities whereas “I feel fatigued” taps into feeling tired, the need for rest, or having reduced energy [3]. Moreover, the results are in line with depictions of fatigue in advanced cancer as a multidimensional and complex experience that involves biochemical, psychological, behavioral, and psychological aspects [15]. Taken together, our findings suggest the items “I have a lack of energy” and “I feel fatigued” are conceptually related, but the items are tapping into different aspects of the fatigue experience. Therefore, we recommend retaining both items on the NFOSI-18 DRS-P.

Next, our qualitative exploration of patient considerations when responding to the item “I am bothered by constipation,” revealed that patients respond according to the perceived severity of their constipation. In addition to reporting they would be more or less bothered if the constipation worsened or improved, patients further elaborated on factors that would increase bother, such as more pain or spasms or an increased frequency or duration, all of which are indicators of constipation severity. Thus, the item “I am bothered by constipation” can be regarded as a measure of constipation severity, a finding that is reinforced by prior reports of a significant association between severity of incontinence and bother [16–18]. Relatedly, Pearman and colleagues recently reported that a similar item, “I am bothered by the side-effects of treatment” is significantly associated with clinician adverse event reporting and patient-reported QOL, which further supports an association between bother and severity [19]. Our findings provide further evidence that bother with constipation is closely related to its severity. This is in line with results from large-scale quantitative studies that report strong associations among bother and symptom severity in other therapeutic contexts [16–20].

Finally, our exploration of the factors considered when patients respond to the item “I am sleeping well,” revealed women considered a number of disease, treatment, and other factors. Nevertheless, because sleep quality is an issue of great importance for cancer patients throughout the disease course, we recommend retaining the item on the NFOSI-18 DRS-P; however, we suggest an alternative scoring option to exclude the item from the DRS-P if it is to unambiguously be referred to as a measure of disease-related symptoms [3, 21–23].

There are three limitations that deserve mention. First, while there was some diversity, the vast majority of cognitive interview participants were White and of non-Hispanic/Latino origin. Second, participants were recruited through a single institution. Both of which limit the generalizability of our findings. Third, although the sample size was determined according to well-established qualitative research practices for cognitive interview sample size [24–26], it is relatively small which could limit the generalizability of the our findings.

## Conclusion

The findings of this cognitive interview study provide further evidence in support of the NFOSI-18 for assessment of PROs in clinical research and practice. Furthermore, the current study adds to prior validation efforts by focusing specifically on the content of the DRS-P, which may be of greatest relevance to clinical trials and ongoing examinations of validity due to changing treatment regimens and symptom profiles over time. While we do not recommend edits to the NFOSI-18, we do propose an alternative scoring option that excludes the item “I am sleeping well” from the DRS-P when used as a symptom index for clinical research. The present findings extend our understanding of patient interpretations of related, yet distinct constructs and patient considerations when responding to ‘bother’ items on PRO measures.

## Data Availability

The interview data generated and analyzed for the current study are not publicly available due to privacy and confidentiality agreements.

## Abbreviations

DRS-P: Disease-Related Symptoms-Physical subscale
ECOG: Eastern Cooperative Oncology Group
FDA: U.S. Food and Drug Administration
NFOSI-18: NCCN FACT-Ovarian Symptom Index
PRO: Patient reported outcome
QOL: Quality of life

## Footnotes

1 Participants were able to choose all options that apply. Therefore, a participant did not have to choose between treatment- and disease-related factors and could endorse both.
